# A Qualitative Study on the Grief of People Who Lose Their Only Child: From the Perspective of Familism Culture

**DOI:** 10.3389/fpsyg.2018.00869

**Published:** 2018-05-30

**Authors:** Yudi Zhang, Xiaoming Jia

**Affiliations:** School of Humanities and Social Sciences, Beijing Institute of Technology, Beijing, China

**Keywords:** familism culture, shiduers, grief, qualitative study, China

## Abstract

Shidu is the Chinese transliteration for ‘losing an only child,’ which indicates the death of the only child in the family. Shidu is a unique social phenomenon resulting from the One-Child Policy implemented in China for several decades. Shiduers are parents who have lost their only child. The grief research scholar Neimeyer (2012) argued that grief research should consider the role of different cultures in the grieving process. Familism culture is a collectivist culture that has a profound effect on Chinese society and is likely to produce a significant effect on the grieving process of shiduers; however, this effect has not yet received systematic attention in research. To explore the effect of familism culture on the grief of shiduers, we conducted semi-structured personal interviews in Beijing, China, with seven shiduers. The study results show that the effect of familism culture on the grief of shiduers includes three levels: cognition, emotion, and behavior. These levels are reflected in a variety of relationships, including relationships with ancestors, the deceased child, the spouse, relatives, Tong Ming Ren (the Chinese transliteration of ‘people who share the same fate’), and the country. The first four types of relationships are reflections of ‘direct familism culture,’ and the latter two types of relationships are reflections of ‘extended familism culture’. The relationships with the deceased child, relatives, and Tong Ming Ren are mainly supportive; the relationship with ancestors is mainly stressful; the relationship with the spouse has a dual nature; and the relationship with the country is contradictory. Over time, shiduers have abandoned the concept of familism culture and have moved toward reducing stress and increasing supportiveness. Psychological professionals, social workers, and government staff may refer to the results of this study to help shiduers obtain support and reduce stress from the described relationships. Specific suggestions are provided in the text.

## Introduction

Previous studies examining the grief of parents who lose their child have primarily addressed two aspects. First, the grief expressed by them has been studied. For example, Michon et al. (2003) used a scale to measure the intensity of grief, whereas Olivas (2013) used a form of textual data to illustrate their state of grief through qualitative research. Second, researchers have continuously attempted to explore the factors that influence the grieving process among them. For example, Rando (1983) found that a previous loss tended to be associated with poorer bereavement outcomes among those who lose their child to cancer, whereas Ronen et al. (2010) interviewed parents who lose their child and found that continuing bonds could promote their grief adjustment. Because of language differences and other barriers, however, the state of grief among Chinese shiduers has rarely received scholarly attention.

Losing a child causes the most painful and lasting grief (Gorer, 1965). China’s One-Child Policy was maintained for approximately 30 years to control the population. This policy ended only recently in 2015. During the several decades of the One-Child Policy, losing an only child became a unique social phenomenon in China. Parents who lose their only child are called ‘shiduers.’ Using census data from 1990, 2000, and 2010 as a basis, Wang estimated that as of 2010, the cumulative number of deceased only children exceeded one million, which corresponds to more than one million families that lost their only child (Wang, 2013). Wang also noted that although the One-Child Policy has ended, the number of future families that will lose an only child will continue to increase for a considerable period of time (Wang, 2016). The results of several studies have suggested that the state of grief of Chinese shiduers is more severe than that of other parents who lose their child. A survey of 226 Chinese women who became shiduers due to earthquakes found that the state of prolonged grief amongst the 116 women who did not give birth again was more severe than that amongst the 110 women who had given birth again (Xu et al., 2014). Furthermore, a cross-cultural study of shiduers in China and parents who lost their child in Switzerland found that those in China scored significantly higher on the prolonged grief scale (Xiu et al., 2016). Therefore, the grief state of shiduers is deserving of research.

A Chinese saying states that ‘losing one’s parent(s) in one’s youth, losing one’s spouse in middle-age, and losing one’s child in old age’ are the three great tragedies in life. The shiduers population undoubtedly experiences an unforgettable grieving process. Grief refers to ‘a primarily emotional reaction to the loss of a loved one through death. It incorporates diverse psychological (cognitive, social-behavioral) and physical (physiological-somatic) manifestations’ (Stroebe et al., 2001). Qualitative studies have found that most shiduers develop an intense grief response in terms of cognition, emotion, behavior, and physiology (He et al., 2014). If this type of intense grief response persists in the long term, prolonged grief can easily develop. Prolonged grief is also called complicated grief, which is a long-lasting and permeating grief response characterized by longing for or persistent preoccupation with the deceased and accompanied by intense emotional pain (World Health Organisation, 2017). According to another survey, the proportion of shiduers who suffered from prolonged grief was approximately 30% (Shang, 2016). Many factors affect shiduers with regard to the suffering they experience from prolonged grief or from the slow recovery process. The effect of culture on this grieving process has been stressed by scholars. For example, the well-known grief research scholar Neimeyer (2012) argued that ‘exporting’ the grief model primarily generated in the United States to other cultures might be inappropriate and that grief research should consider the role of different cultures in the grieving process.

Studies on the relation between the grief of shiduers and their culture are rare because shidu is a unique social phenomenon in China. Recently, relevant studies have discussed the effect of culture. For example, in Chinese culture, participants in funeral rites are usually a group formed by ties of blood and geography (Jia, 2010). The grieving process for shiduers is affected by the negative influence of ‘stigmatization’ from traditional Chinese culture (Xu, 2014; Zheng and Lawson, 2014). The ritualistic behavior of grieving for shiduers has unique Chinese cultural connotations. For example, according to traditional Chinese customs, some shiduers hold a sacrificial ceremony for the child every 7 days for 49 days after the child’s passing (He et al., 2017). However, the objectives of these studies were not to systematically examine the effect of culture. These studies provided only partial information to understand the grief of shiduers from a cultural perspective and lacked in-depth studies of a particular effect of culture. Therefore, the question remains: for shiduers in China, which type of traditional Chinese culture has the most profound effect on their grieving process?

China is a country deeply affected by the collectivist culture. Among the numerous types of collectivist cultures, familism culture has an especially profound effect on Chinese society. Familism culture refers to a type of ‘ingroup collectivism’ in which family members attach primary importance to the family (Schwartz, 1990). In China, however, the word ‘family’ refers not only to close relatives, such as parents, spouses, and children, but also to a social organization formed by ties of blood or marriage that encompasses nephews, aunts, and other relatives (Chinese Academy of Social Sciences, 2012). The famous Chinese social psychologist Guoshu Yang generalized the social orientation of Chinese people into four characteristics: family orientation (the most important characteristic), relationship orientation, authority orientation, and other orientation (Yang, 2004). In his book *The Essentials of Chinese Culture*, the famous Chinese thinker Liang (2011) noted, ‘Everyone knows that the family is a particularly important relationship in the lives of Chinese people; Chinese people value such relationships much more than Westerners.’ The formation of this type of culture is attributed to the family having served as the basic unit of social structure and function in China’s traditional agrarian society for several 1000 years. The core of familism culture is a set of relationships of rights and obligations that attach primary importance to the interests of the family in all things, including at the three levels of cognition, emotion, and behavioral tendency. An example at the cognitive level is the importance placed on the continuance, solidarity, and reputation of the family. Examples at the emotional level are a sense of belonging, a sense of responsibility, and a sense of security. Examples at the behavioral tendency level are reproducing one’s offspring, interdependence, and striving for the family (Yang, 2004). Bowen (1978) proposed the family systems theory, which considers the family as a functional organic whole. Subsequently, scholars argued that grief is not an individualized experience, and the family, as the most important social organization for an individual, exerts important influences on the grieving process (Detmer and Lamberti, 1991; Kissane and Bloch, 1994). Within the familism culture of China, many grieving activities are shared with family members. For example, making sacrifices and cleaning tombs with the whole family during the Qingming Festival is a way for the family to connect with lost loved ones (Jia, 2005). Thus, against the cultural backdrop of familism, how might the grieving process of shiduers in China be affected?

There are many expressions in familism culture regarding carrying on the familial line. All are directly related to shiduers, such as, ’There are three unfilial acts, of which having no posterity is the greatest’ and ‘more children will bring more happiness.’ Therefore, for Chinese shiduers, ‘shidu’ means more than just losing their only child. It means losing the hope of having descendants and carrying on the family line, and the continuity of the family line in familism culture is seen as a duty to the ancestors. This unrealized and unfulfilled duty may have negative effects on the grief process of shiduers. In the grieving process of shiduers, familism culture may play an important role. For example, psychoanalysts emphasize the positive role of a container in the grieving process and suggest that other people can facilitate the completion of meaning making and the integration of the life narrative by containing the grieving person’s emotions (Rolls, 2007). Familism culture advocates mutual support and help among family members. Does this support allow the family to become a large container for the grief of shiduers? Another survey found that 85.8% of shiduers felt inferior (Fang, 2015). Is this feeling caused by the enormous psychological burden experienced by shiduers due to the importance that familism culture places on reproducing offspring? From a more optimistic perspective, however, the bond and harmony among relatives in familism culture may be beneficial for the grief process of shiduers. To summarize the above research, the present study poses the following questions. How does familism culture play a role in the grieving process of shiduers? Specifically, through which channels does it play a positive role, through which channels does it play a negative role, through which channels does it play both positive and negative roles, and how are these roles played?

This study seeks answers to these questions to increase understanding of the grief of shiduers in China from a cultural perspective, both for the enrichment and development of theories on grief and in the hope of providing useful suggestions to psychological and social services aimed at helping shiduers.

## Materials and Methods

### Study Context and Design

This study is part of the project ‘Research on the Effect of Familism Culture on the Grief and Mental Health of Shiduers.’ The data for this report came from the qualitative study portion of that project, which was based on a phenomenological perspective and used the semi-structured interview method to survey seven shiduers individually in hopes of understanding the impact of familism culture on their grief. The implementation of this study was approved by the Ethics Committee of the School of Humanities and Social Sciences at Beijing Institute of Technology. All interviewees in this study signed a written informed consent form.

### Study Population, Sampling, and Recruitment

We first performed volunteer service at multiple service agencies that help shiduers in Beijing, China, including the ‘Homestead for the Psyche’ for shiduers established in three subdistricts (a subdistrict is the most basic level of government in Chinese cities) and a self-service organization for shiduers. The subdistrict ‘Homestead for the Psyche’ had full-time government staff and specialized venues, was open only to shiduers within the area of jurisdiction, and held activities such as singing, dancing, handicrafts, and culinary arts on workdays. The self-service organization for shiduers existed in the form of a WeChat (an instant messaging app) group. The members were shiduers from all areas of Beijing, and the group leader organized occasional gatherings and activities or organized group members to help shiduers who were having difficulties. Based on the objective of this study, we used a non-probability purposive sampling approach. That is, by participating in activities, we observed which of the shiduers were most likely to provide abundant information for the research project and then individually sent invitations to these shiduers to participate in the study. When sending invitations for the study, we explained to the invitees the study’s objective, content, possible risks, and the rights of the participants. During the interviews, we continually summarized the themes of the interview content. When every theme had reached saturation, no new invitations for the study were issued. At this time, a total of seven interviewees had participated.

### Participants

We sent 12 individual interview invitations; five people refused, and seven people accepted. These seven participants included two males and five females ranging in age from 51 to 74 years with a mean age of 62.7 years. The deceased children of three participants were boys, and the deceased children of the other four participants were girls. The age at which the children passed away was between 6 and 33 years, with a mean age of 24.3 years. The duration since the children had died was between 0.5 and 25 years, with a mean duration of 11.6 years. See **Table 1** for more details on the corresponding relations among this information.

**Table 1:** Table of basic information on the interviewees.

Interviewee	Gender	Age	Gender of child	Age of child at death	Time since the child’s death (Year)
A	Male	67	Female	29	10
B	Female	74	Male	29	20
C	Female	64	Male	33	5
D	Female	57	Female	26	5
E	Male	68	Female	22	16
F	Female	58	Male	6	25
G	Female	51	Female	25	0.5

### Data Collection

The interviews were conducted between June and September 2017. In accordance with the wishes of the interviewees, three people were interviewed at their homes, and four people were interviewed in a quiet room at the activity venue for shiduers. We used semi-structured interviews in which a summarized interview outline was prepared in advance, and detailed inquiries were made depending on the interviewee’s answers in the course of the interview. In the first step, a detailed explanation was given to the interviewee on the study’s objective, content, possible risks, and the rights of the participants, including the information that the interviewer was about to begin recording. If the interviewee agreed to be recorded, a written informed consent form needed to be signed. In step two, it was inadvisable to begin directly with the themes because the interview themes could easily trigger negative emotions. Therefore, the interviewer first exchanged pleasantries with the interviewee and chatted about topics that were easier to discuss, such as the participant’s hobbies. In step three, after exchanging pleasantries and obtaining the consent of the interviewee, the interviewer formally began the interview with four summarized questions in the interview outline: (1) After you lost your child, how do you feel your family influenced you in various aspects, including favorable or unfavorable aspects? (2) Do you feel that the influence you just mentioned was related to traditional Chinese culture, especially traditional culture involving family? (3) In the process of mourning your child and slowly processing the pain in your heart, do you feel that some things in traditional Chinese culture hindered you? If so, what were they? Do you feel that some things in traditional culture helped you? If so, what were they? (4) Is there anything else you want to tell me? In step four, the formal interview ended, and the interviewer observed the interviewee’s emotions, asked whether help was needed, and informed the interviewee that relevant resources could be recommended if professional assistance was required. The interviewer paid the interviewee 100 Renminbi (RMB; approximately 16 USD) as remuneration for participating in the study and as an expression of gratitude. The interview duration was 63–120 min, and the total interview duration for the seven people was 585 min.

### Data Analyses

In this study, thematic analysis was used to analyze the data. The analyst in the following text refers to the first author of this study, YZ. First, the analyst performed a verbatim transcription of the audio recordings obtained from the interviews. Transcription is an important means of controlling the quality of qualitative research (Steinke, 2004). During transcription, the analyst strived to maintain the original meaning by completely and accurately recording the words spoken by the interviewees, the pauses, and the tone. After the transcription was complete, a transcription text with a total count of 86,696 Chinese characters was obtained. Then, referring to the suggestions of Braun and Clarke (2006, 2012), the analyst took the following steps to analyze the data. (1) A full-text reading of the verbatim transcription text was conducted. While reading, the analyst recorded initial thoughts with regard to coding. (2) The analyst generated initial codes based on the text and obtained 1,440 codes. (3) The analyst wrote the name of each code on separate pieces of paper and attempted to organize them into candidate themes. Five candidate themes were created: ‘relationship with ancestors,’ ‘relationship with the deceased child,’ ‘relationship with loved ones,’ ‘relationship with Tong Ming Ren,’ and ‘relationship with the country.’ (4) The analyst discussed the candidate themes with XJ, another author of this study who is a psychologist and has been engaged for many years in research related to the loss and grief of shiduers. The authors agreed that it was appropriate to divide the theme of ‘relationship with loved ones’ into ‘relationship with spouse’ and ‘relationship with relatives.’ In Chinese culture, the spouse and relatives are both considered loved ones, but further discussion between the two authors concerning the text and coding clearly differentiated the shiduers’ relationships with their spouses from those with their relatives. Therefore, these groups were divided into different themes. The two authors reached a high degree of consensus after dividing this theme based on multiple discussions. (5) The themes obtained from this study as well as the definition and description of the themes were sent to two participants of this study with a request to assess whether the division of themes was appropriate. Both participants expressed a high degree of approval regarding the division of the themes. (6) The study report was written based on the developed themes.

## Results

Starting from the perspective of familism, this study found that the grief of the shiduers was primarily embodied in various relationships, including those within the following six themes: relationship with ancestors, relationship with the deceased child, relationship with spouse, relationship with relatives, relationship with Tong Ming Ren, and relationship with the country.

### Theme 1: Relationship With Ancestors

In familism culture, producing an offspring is an important responsibility, ‘continuing the ancestral line’ is an obligation to the ancestors, and having no posterity is seen as unfilial. The interviewees who lost their only child were deeply affected by the traditional culture of familism and could elaborate on their understanding of this culture by citing idioms or traditional art forms, with examples provided from Interviewees B and E. Interviewee B called these idioms ‘auspicious sayings,’ indicating that in his understanding, the opposite situation was ‘inauspicious.’

*Ah, ‘more children will bring more happiness’ is what old people used to say in the past. For example, someone says (asks), ‘Why did you give birth to so many children?’ (He answers) ‘who knows which cloud bears rain?’ He is saying that I have many children, so one is bound to be successful. …If you do not have children…it means it is a misfortune of a family. ‘There are three unfilial acts, of which having no posterity is the greatest’ …there are three unfilial acts, and ‘having no posterity’ means you do not have descendants; it is the greatest unfilial act.* (Interviewee B, woman, age 74, child age 29).

*Look at that little decoration; so many children are climbing on the body of the big Maitreya Buddha. There are many auspicious sayings, like ‘more children will bring more happiness.’ A 100 children painting depicts a lot of children dressed in ancient costumes playing all kinds of games…then, there’s that other auspicious Chinese saying that is included on buildings, ‘get a pomegranate.’ Does a pomegranate actually look good? It just has symbolic meaning because it has many seeds (in Chinese, ‘seed’ sounds the same as ‘son’), so more children will bring more happiness.* (Interviewee E, man, age 68, child age 22).

Although shiduers expressed their understandings, changes occurred over time in ideas related to producing offspring. The people of this generation who had lost their only child could clearly understand the connotations of the concept of ‘continuing the ancestral line,’ but they did not necessarily personally uphold such concepts. In the interviews of this study, Interviewees B, E, and G expressed disagreement with this type of traditional culture and indicated they could feel changes occurring in the culture over time.

*‘There are three unfilial acts, of which having no posterity is the greatest’; this was for the generation before me. They obviously believe in that saying. As for me, I received a new education, so I do not believe this idea. I feel it is fine that (this concept is) changing (with the times) and has become weaker. In the generation after me, there are dual-income, no kid (DINK) households.* (Interviewee B, woman, age 74, child age 29).

*Like, in this type of situation where there is only one (child), once she was gone—we were originally particular about having a boy or a girl—only a boy can continue the ancestral line. Now, there is only one; since there is no choice, it doesn’t matter if it is a boy or a girl….I do not put much stock in this so-called idea of continuing the ancestral line. It is your only child, in any case.* (Interviewee E, man, age 68, child age 22).

*(Speaking of ‘there are three unfilial acts, of which having no posterity is the greatest’) this thing is basically nonsense…perhaps the last generation was a bit more traditional, and we may be slightly better at not sticking to the saying. In terms of this thing of descendants, I am not particularly concerned with having descendants.* (Interviewee G, woman, age 51, child age 25).

### Theme 2: Relationship With the Deceased Child

The approach to mourning and burial is an important part of familism culture (Yang, 1992). With regard to the place of mourning and burial, a strong relationship between life and death is achieved among family members through burial at the same or a nearby place. Many shiduers buried the child next to their own parents (that is, the child’s grandparents) or their own spouse, as Interviewee C did. Some shiduers also chose or attempted to choose a cemetery with multiple plots in preparation for the shiduer’s burial at the same gravesite as the child, as Interviewees D and G did. In addition, the things left behind by the child (such as photographs) served as symbols of the child. Shiduers such as Interviewee C put the left-behind things in their own home and sometimes evoked emotional attachment with the child through the left-behind items.

*I go (to the cemetery) to visit every year during Qingming Festival. I go once a year and visit my spouse also; they are together.* (Interviewee C, woman, age 64, child age 33).

*After she left, I insisted on having it…it is so expensive here! (Referring to a local cemetery that charged very high fees.) I must put my daughter here even though I have to spend my entire fortune. Since my parents are here, we are very close…she has a double plot, and I will surely be beside her! She is waiting for me. I feel I have no regrets, and I still want to be with her.* (Interviewee D, woman, age 57, child age 26).

*At the time, I originally planned to have all three of us buried together. I said, let us buy a site with three plots, but the site was not available, so we could only let her have a single plot.* (Interviewee G, woman, age 51, child age 25).

*I look at photographs to find the feelings when he was a small child and recall the memories.* (Interviewee C, woman, age 64, child age 33).

Prior to the rise of research on continuing bonds, Western culture tended to deem communication with the dead through dreams and visual or auditory experiences a psychiatric symptom (Olivas, 2013). However, Chinese familism culture holds a completely different view. For example, a saying exists in familism culture concerning the ‘spirit in heaven’ (Yang, 2000), which can communicate with family members who are still living so that the influence of the deceased individual does not disappear. This ‘spirit in heaven’ mostly refers to one’s ancestors; however, this study found that the spirit in heaven of a child had a major influence on the shiduers. Interviewee A felt that his motivation to live was to be worthy of the deceased child. Interviewee B thought that she had to pull herself together because only by being alive and well would there be a person in this world who missed the child. Interviewee G believed the child went to ‘another world,’ one not completely separated from the real world in which the shiduers lived, and the two worlds were linked through some sort of mysterious channel; therefore, the child communicated with her through dreams, hallucinations, and other activities. Interviewee D believed that after she dies, she will go to the world where her child is and meet her.

*I have to live and be strong. The most important reason is that I (must) be worthy of my dead child.* (Interviewee A, man, age 67, child age 29).

*How did I come around at the time? I thought that if we were both in good health, then even in this world, there would still be people who cherished his memory, who still thought about him. If our health was bad and we die, then it would be completely over; there would be no one to think about him. In this way, we both came around later. I had to pull myself together.* (Interviewee B, woman, age 74, child age 29).

*On the second day, she returned in a dream…later that night, she embraced me, and the two of us slept for a night. It was equal to saying a kind of goodbye; it seemed similar to saying goodbye.* (Interviewee G, woman, age 51, child age 25).

*I was on an outbound flight and reached the skies over Montreal. I was sitting next to the porthole on the airplane and suddenly heard my daughter calling me, calling with vigor, ‘Mom! Mom!’ I was a bit stunned at the time when I heard the first sound. Afterward, she called two more times. It was unlike her usual (soft) calling, ‘Mama, Mama.’ Hers was a very loud shout, ‘Mom!’, as if afraid I could not hear. I slowly turned toward the porthole and looked outside. I said, ‘Are you calling for me? Are you calling for Mama?’* (The interviewee cried and became silent for 40 s) *I said, ‘Mama has come. I will bring you back.’ Then she did not call out anymore.* (Interviewee G, woman, age 51, child age 25).

*In the future, I will be with my daughter. We will meet each other there. She is definitely waiting for me. She thinks that the later I come the better. She is fine there. I do not need to rush there.* (Interviewee D, woman, age 57, child age 26).

### Theme 3: Relationship With Spouse

This study found that the obligation to produce offspring and family unity proposed by familism culture generated conflict in the shiduers. After the child passed away, the shiduers faced choices in terms of how to handle their relationships with their spouses. Should they maintain a good relationship with their spouses in accordance with the obligation of family unity, or should they vent emotions in the home because of their inability to satisfy the obligation to produce offspring? Should they go so far as to continue to produce offspring through divorce and remarriage? The spousal relationships of different shiduers may go in different directions. Some people are affected by the grief of losing an only child and become irritable and prone to conflicts with their spouse, particularly soon after losing an only child, as in the situation mentioned by Interviewee A. None of the interviewees in this study divorced due to the death of the child, but Interviewee F lashed out when speaking of the phenomenon of men who lost their only child divorcing and remarrying to produce another offspring. Shiduer spouses who supported each other and helped each other in simple ways were not lacking. Interviewee F talked about the feeling of ‘depending on each other for survival’ with her husband. Interviewee C’s husband passed away a dozen years ago; she personally witnessed the situation of people with spouses who had each other when they lost their only child, and she sighed with heartfelt emotion and with some envy.

*After losing an only child, the first obstacle for the young parent is marital crisis. Once the child dies, both people feel miserable. People lose their reason when they are miserable. Sometimes, you may not find me as pleasing to the eye, and I also may not find you as pleasing to the eye. There is no other place to vent, so one takes things out on the other.* (Interviewee A, man, age 67, child age 29).

*You don’t want your spouse, right? You trade her in for a younger one to give birth to a child for you. How old are you now? Speaking based on my age, over 50. You want another one. You live another 30, can you live for 30 more years? In the end, you get a widow and a child. Just when (you) are needed, you are gone. Are you not cheating others? I disapprove of this. I feel there is no need for this! Having gone through thick and thin together for so many years, this is the time when you are needed; you have no conscience!* (Interviewee F, woman, age 58, child age 6).

*No matter how you look at it, having a spouse is having someone to rely on. You lay there and can’t move; he cooks for you and talks with you. You don’t feel well, so let’s go to the hospital to be seen. At least there is someone who cares about you. Be considerate to one another. If the couple is considerate to each other and cares about each other, then it is not the same as with me (my situation), where I am alone.* (Interviewee C, woman, age 64, child age 33).

*It’s okay, okay. After all, we are an old couple, what can we do? What do you do if you do not depend on each other for survival again? You can only depend on each other for survival. Otherwise, wouldn’t it be worse? It would be lonelier. That is, if you are sick, someone will go with you, and it is fine if there is no major illness. There are two people to care about each other and to discuss what to do when something comes up.* (Interviewee F, woman, age 58, child age 6).

### Theme 4: Relationship With Relatives

The relatives discussed here refer to family members other than ancestors, the deceased child, and the spouse. Relationships with relatives are established through ties of blood or marriage. Under the influence of the concept advocated by familism culture that ‘family members must stick together,’ the relatives of shiduers often gave the shiduers some support but were also subject to the influence of the concept in familism culture that ‘having more children and grandchildren is lucky; having no descendant is inauspicious.’ Relatives often believe that shidu is extremely unlucky; it cannot be mentioned to avoid upsetting the shiduers. Thus, on the surface, the support of relatives is often not at an emotional level. For example, they may provide material help or help with activities or they may visit the child’s grave together, but the motivation and emotions for providing these types of support often were not expressed, as described by Interviewees A, B, and E.

*(With relatives) sometimes we visit each other; sometimes they come over to see us. For example, if I were sick and hospitalized, they would all come to the hospital to visit me. This is care, right?* (Interviewee A, man, age 67, child age 29).

*Although they didn’t say it clearly, I can understand (their meaning): ‘although your family is incomplete, you still have an intact big family.’ I feel it is this way. For example, my birthday, in the past, they have never celebrated my birthday, and also my husband’s birthday. (After the child passed away,) they organized a birthday celebration for us…They said to me, ‘Tell us right away if you have anything going on! You have to tell us!” Don’t hold out and keep it to yourself, especially things you need help with. Don’t treat us like outsiders’.... This issue is not discussed at all in interactions with relatives…Everyone talks about his/her work, life, family life, not about this event in our family. No one talks about it…I feel the way everyone treats us is a type of respect. They don’t want to open your scar.* (Interviewee B, woman, age 74, child age 29).

*The child is buried in Wanan (name of a cemetery). I must go every year, and the relatives also go. Sometimes we go together; sometimes they go on their own. I’m the eldest in my family…at the time, the effect was deeper because I was the first among several brothers to have a second generation. Afterward, it lessened, and now I’m also less affected. The relatives don’t bring up this matter, and it’s slowly fading.* (Interviewee E, man, age 68, child age 22).

### Theme 5: Relationship With Tong Ming Ren

‘Tong Ming Ren’ in this study refers to other shiduers. Most shiduers like to be with Tong Ming Ren and are able to feel family like comfort from this type of togetherness. Although Tong Ming Ren are not each other’s family members, the mode by which they get along is very similar to that of family members. In Beijing, the government provides shiduers with specialized activity venues, most of which are titled with the name ‘XX Homestead.’ In the narratives of shiduers, ‘homestead’ and ‘loved ones’ were words that appeared often, suggesting that the Tong Ming Ren were quasi-family members without ties of blood or marriage, as indicated by the narratives of Interviewees C, D, and F.

*To us, these people who lost the only child, having a homestead seems to evoke a kind of feeling like being at home.* (Interviewee C, woman, age 64, child age 33).

*I am now consciously increasing the frequency (of interactions with Tong Ming Ren). Why? I feel comforted.* (Interviewee D, woman, age 57, child age 26).

*All of us voluntarily joined this group. After joining, everybody helps each other if anything happens. It is just like being a volunteer. If something happens, everybody would especially remember with concern. After all, since our hearts have suffered trauma, we can understand each other. Perhaps at the time when she was irritable, she would say something we didn’t like to hear, but we could all understand it was due to being troubled by the wounds. We could understand feeling some irritability, but people in general didn’t understand. When all was said and done, because she saw her own loved ones after coming here, that is, seeing us come, these Tong Ming Ren, it was just like her loved ones, she naturally…could not help it. But after it was over, she knew…after getting under control, she would apologize on her own, knowing that, ‘I did something wrong.’ Some people could not stand it; ‘You have mental problems.’ No! It was because she had wounds for many years that troubled her. In actually seeing loved ones, she could vent her temper a bit; this was the case… (Speaking of the name ‘homestead,’) yes, I treat it as my parents’ home.* (Interviewee F, woman, age 58, child age 6).

### Theme 6: Relationship With the Country

In Chinese, ‘country’ is pronounced ‘guo jia’; ‘jia’ means ‘family.’ Chinese people often speak of the relationships with their own families and the country as relationships with the ‘small family’ and the ‘big family,’ respectively. Therefore, the country also has a cultural connotation of family to Chinese people. The loss of the only child is related to the state policy; therefore, the feelings of shiduers toward the country were relatively conflicted. On the one hand, resentment and loss were expressed; on the other hand, reliance on the country was very much desired, similar to children at home who want to rely on their parents, as stated by Interviewees F and G. For this generation of shiduers, obedience to the state had been taught since childhood, and the shiduers were similar to children who were afraid to disobey their parents, as stated by Interviewee C. When government staff organized group activities for shiduers, the shiduers were proud of showing ‘conscientiousness’ and not being ‘mischievous,’ as stated by Interviewee F.

*In my time, having one was being advocated already. They said it was good to give birth to one. You had resentment…that was the system set by the state. We dared not have it, and what could you do if you had it? In any case, at the time we just listened to the leader on everything, listened to the state.* (Interviewee C, woman, age 64, child age 33).

*In general, like in our case, if the subdistrict organizes activities or something where we can move around in most conditions, we would participate. Because, after all, to put it plainly, the party and the government are quite concerned about this, so everyone is willing to go. Also, after going, these people are very conscientious. If you say, let’s gather, they would all return at the appointed time, unlike those mischievous types who don’t return at the appointed time. No, they are very obedient and very willing to participate in this activity.* (Interviewee F, woman, age 58, child age 6).

*(Speaking of the lack of assistance) People like us have a sense of loss; he is not complaining, he is lost. At the outset, people said, I will make a contribution to the country; if the country doesn’t let us have it* (a second child), *then we are willing to not having a second child; it is fine either way. Except now, when we need the country to manage us, there is no one. We are old and need someone to take care of us, but there is no one. He has a special sense of loss. Once the sense of loss is great, why can’t he complain?* (Interviewee F, woman, age 58, child age 6).

*There is hate, but there is also a desire for reliance because, how to say it after all, I am certain that in the end we still have to rely on the government. Personal power is still nothing…state policy, to the entire group that lost an only child, is truly beneficial to the whole group.* (Interviewee G, woman, age 51, child age 25).

*In general, if you say you question the country’s policies, then it is a bit… hard to say... because the [One-Child Policy] officially began in the 1970s and persisted through the 1980s and 1990s. [The government] being open to having a second child started only now. So, I say that the generations born during the 1980s, 1990s, 2000s, and 2010s are full of only children. It may be a bit better for the generation born in the 2010s; you might have two [children] in 2010. Therefore, I say in terms of three generations, for sure up to the present, there are still many shidu families… (Interviewee G*, woman, age 51, child age 25*).*

## Discussion

### Presence of Familism Culture in the Relationships of Shiduers

Xiaotong Fei, a well-known Chinese sociologist, used the following analogy to describe the closeness of interpersonal relationships among Chinese people: when a stone is thrown into water and the entry point is used as the center, rings of ripples are generated from the inside to the outside; the distance from the ripple to the center of the entry point represents the closeness of the relationship (Fei, 2012). To many Chinese people, family members are the ripple closest to the entry point. This is the deep influence of several thousand years of China’s agrarian society. In traditional Chinese society, the family is the basic unit of structure and function. This understanding differs from Western society, in which the individual is the basic unit (Yang, 2000). In this study, familism culture was embodied in the lives of the shiduers in a variety of relationships, including relationships with ancestors, with the deceased child, with the spouse, with relatives, with Tong Ming Ren, and with the country. These relationships reflected the grief of the shiduers from different angles. Losing an only child is a traumatic event resulting in the loss of an important relationship, which can damage an individual’s sense of security, sense of control, self-esteem, and sense of trust in the environment (Herman, 1992). ‘Relating to others’ was found to be an important indicator of post-traumatic growth (Tedeschi and Calhoun, 2004), suggesting that grief caused by the loss of a relationship needs to be rehabilitated with other relationships.

In the relationships of the shiduers with their ancestors, the deceased child, the spouse, the relatives, Tong Ming Ren, and the country, the first four types of relationships are relationships with living or dead family members. In familism culture, death does not affect an individual’s position as a family member; the saying goes, ‘born as a member of a certain family, die as the ghost of a certain family’ (Wu, 2011). Additionally, death does not affect continued concern for the family, as indicated by the common Chinese saying, ‘the spirits of the ancestors in heaven’ (Yang, 2007), which suggests that the ‘spirit in heaven’ sees whether the behavior of the living is appropriate and may both protect and punish living family members. These four types of relationships do not exceed the connotations of familism culture and may be called ‘direct familism culture’. In the past, discussions on the connotations of familism culture were usually within the scope of ‘direct familism culture.’ For example, Guoshu Yang stated that the connotations of familism culture included unity and harmony, reproducing for the family, and making the family financial situation prosperous (Yang, 2004). This understanding contrasts with the results of the present study in which unity and harmony are the focus for relationships with relatives, whereas reproducing for the family and making the family financial situation prosperous are mainly obligations to the ancestors. In addition, although the relationships with Tong Ming Ren and with the country cannot be covered in a strict definition of familism culture, the nature and the emotions contained in these two types of relationships are quite similar to the first four types of relationships and may be considered an ‘extended familism culture.’

One case study argued that the relationship between shiduers and Tong Ming Ren can act as a partial substitute for the deceased child’s affections (Chen, 2014). In this study, interviewee F directly called Tong Ming Ren ‘loved ones,’ which is a vivid statement. With regard to the relationship with the country, due to the influence of 1000s of years of ‘loyalty and filial piety culture’ in China, a type of conflict is reflected in the emotions of the shiduers toward the country. On the one hand, the One-Child Policy is one of the reasons for shidu, and shiduers may harbor resentment. On the other hand, shiduers have often received patriotic education since they were young and see ‘having utmost loyalty to the country’ as their obligation. Although ‘having no descendant’ is a type of unfilial act against the ancestors, in the same vein, a saying exists in the *Book of Filial Piety* linking ‘substitute filial piety with loyalty to the country’; the saying unites being loyal to the country with being filial to parents. Because the country is the ‘big family’ and the individual family is the ‘small family,’ ‘giving up the small family for the big family’ is an act praised by Chinese culture. At this level, the unfilial act of shiduers against the ancestors can find a basis for forgiveness in the culture. Because China is a society that places importance on relationships, Chinese people often have an interdependent self-construct in which the self is defined through relationships with important objects (Markus and Kitayama, 1991). Therefore, understanding the described relationships is conducive to a deep understanding of the grief of shiduers and to understanding how shiduers view themselves after their loss.

### Support and Pressure of Familism Culture on the Grief of Shiduers

Research has shown that shiduers not only need to handle the pain of loss but also face pressure imposed by the social context (Wang et al., 2017). However, this research did not indicate how pressure from the different areas differs. A study found that social support and the subjective sense of well-being in shiduers showed a significant positive correlation (Yao et al., 2016), but that study did not indicate how support from the different areas differed. The effect of the six types of relationships on the grief of shiduers found in this study is mainly reflected in two aspects: support and pressure.

The relationships with the deceased child, with relatives, and with Tong Ming Ren are mainly supportive. Shiduers gain strength by staying connected with the child; for example, an interviewee in this study mentioned burying the child beside her future gravesite. Furthermore, making funeral arrangements according to the interviewees’ own beliefs helped them gain a sense of control over life (Jia, 2010). Relatives provided some material or transactional support. Tong Ming Ren can provide emotional support on the basis of mutual understanding. Therefore, the deceased child, relatives, and Tong Ming Ren provide support to shiduers from different angles. In the introduction portion of this paper, we posed a question: familism culture advocates mutual support and help among family members; does this support allow the family to become a large container for the grief of the shiduers? The results of this study suggest that support from the first three types of relationships may provide a huge container. For example, the results section presented Interviewee F’s mention of a person who lost her only child and vented her temper on the Tong Ming Ren, but the Tong Ming Ren assumed a very tolerant attitude. According to psychoanalytic theory, such a container is conducive to shiduers, allowing them to rebuild a sense of security and a sense of trust in the environment (Symington and Symington, 1996). Another example is Interviewee G’s mention of communication with the deceased child through her own dreams and auditory experiences. One study found that after-death communication (i.e., communication with the deceased child through visual, auditory, or dream experiences) played a positive role in the grieving process of shiduers (Olivas, 2013). In another example, Interviewee B mentioned that her relatives repeatedly reminded her to ask for any help that she needed; this situation increased the availability of her social support. One study showed that the availability of social support amongst the elderly positively predicts their social functioning (Guo et al., 2017). Furthermore, according to the Selah grieving model of Cacciatore (2012), providing community service, helping others go through the grieving process smoothly, and other acts that require social functioning are behavioral indicators of the adaptability of the grieving person. A study found that Chinese people experienced stronger grief and worse health than Americans did in the initial grieving period, but the feeling of pain and self-reported health conditions of Chinese people were better than those of Americans after 18 months (Bonanno et al., 2005). This finding may be due to the positive effect of these supportive relationships.

The relationship with ancestors is mainly stressful. This type of pressure originates from the emphasis in familism culture that posterity must fulfill obligations to the ancestors by ‘reproducing offspring.’ If the obligation is not fulfilled, those who fail to continue the lineage feel ashamed, thereby causing a decrease in the level of self-esteem in shiduers (Hou, 2015). The relationship with the spouse has a dual nature. On the one hand, some shiduer spouses provided each other with concern and care in accordance with the requirement for family unity and harmony requirement within familism culture, especially spouses who became shidu for longer periods of time. On the other hand, the propensity for familism culture to condemn a lack of offspring prevented the shiduers from recovering from their deep sorrow and made it easy for contradictions to arise between certain shiduers and their spouses, especially those who had not been shidu for long. The relationship with the country is contradictory because support and pressure coexist. On the one hand, the concern of the country for shiduers makes them feel warmth and a desire for reliance on the country, similar to a child’s reliance on adults at home. On the other hand, most shiduers feel this type of support is insufficient. Therefore, although they may feel the country’s assistance is inadequate, pressure comes from concerns about how to live well in the future. In addition, because of the emphasis that familism culture places on parental authority, most shiduers have difficulty expressing dissatisfaction with the government (the ‘big parent’). For example, Interviewee G discussed the continuation of the One-Child Policy for several decades, which was associated with numerous shidu families. Emotions of dissatisfaction were present, but she did not directly criticize the government; rather, she spoke through euphemisms. This question was also raised in the introduction portion. For example, one survey found that 85.8% of shiduers felt inferior (Fang, 2015). Thus, the question arises: is this feeling caused by the enormous psychological baggage placed on to shiduers by the importance familism culture attaches to producing offspring? No obvious ‘stigmatization’ components were found in this study in this stressful relationship with the country. This type of situation may be due to the self-stigmatization of shiduers; however, because the interviewees of this study all lived in a relatively inclusive and open major city, specific causes require further study.

The study results suggest that shiduers and their family members are abandoning some aspects of familism culture. For example, although the interviewees in this study were very clear on the obligations required by familism culture to ancestors, such as ‘continuing the ancestral line,’ these shiduers no longer upheld all of these concepts and were therefore subject to less pressure in this respect. Additionally, in this study, many of the interviewees reported support from relatives, and no interviewees reported discrimination from relatives. This finding suggests that over time, the stigmatization experienced from ‘having no descendant’ has decreased.

### Recommendations for Psychological and Social Professionals and the Government

Psychological and social professionals and the government may refer to the results of this study to help shiduers obtain support and reduce stress from the described relationships. For example, the cultural connotations of continuing the ancestral line can be discussed with shiduers, who can be encouraged to abandon this type of cultural expectation. They can be helped to establish a positive connection with the deceased child that allows them to hold the child in their hearts and to bravely face a new life. When necessary, counseling or family therapy for partners who lost their only child can be provided to improve the interpersonal skills of the partners to make the relationship between them more supportive. They can be counseled in communication skills that help them in relationships with relatives and that encourage them to ask for help from relatives to resolve difficult issues in their lives. The shiduer population can be helped by the establishment and improvement of self-service organizations, that is, the group of ‘Tong Ming Ren.’ The government needs to increase assistance efforts and care for shiduers, thereby allowing shiduers to truly feel warmth from their ‘big family,’ the country.

### Limitations

All interviewees in this study came from Beijing, a mega-city with an inclusive and open cultural atmosphere. The support and pressure that shiduers in Beijing experienced might differ from that in small cities and rural areas. Therefore, caution is needed in interpreting the results of this study, especially with regard to the support and pressure experienced in the six described types of relationships.

Chinese familism culture views the status of men as more important and emphasizes the man’s authority in the household (Yang, 2000). Because of the cultural value of this role, men are often ashamed to place themselves in a position of weakness and seek help. As such, far fewer male shiduers than female shiduers were willing to participate in the activities of the service agencies. Based on the observation of this researcher (no accurate count was made), the number of male shiduers who participated in activities was approximately one-fifth that of female shiduers. The sampling location of this study was a service agency for shiduers; therefore, finding male shiduers as subjects presented some difficulties. In fact, only two of the seven participants of this study were men. In addition to this gender imbalance, another limitation of this study was the heterogeneity of the age range for the deceased children of the participants in this study (6–33 years) and the length of time since the child’s passing (0.5–25 years).

## Conclusion

In this study, six important relationships were identified in the grieving process of shiduers: relationships with ancestors, with the deceased child, with the spouse, with relatives, with Tong Ming Ren, and with the country. The first four types of relationships are relationships with family members, embodying the effect of ‘direct familism culture’ on the grief of shiduers. Although the latter two types are not relationships with family members, they have characteristics of the relationships with family members, embodying the effect of ‘extended familism culture’ on the grief of shiduers. Among these relationships, the relationships with the deceased child, with relatives, and with Tong Ming Ren are mainly supportive; the relationship with ancestors is mainly stressful; the relationship with the spouse has a dual nature; and the relationship with the country is contradictory. Over time, shiduers have gradually abandoned the influence of familism culture, causing the effect of familism culture on the grief of shiduers to be mainly positive. Understanding these relationships of shiduers is useful to psychological and social professionals and to the government to help shiduers obtain support and reduce stress, thereby enhancing their quality of life.

## Ethics Statement

This study was carried out in accordance with the recommendations of the Ethics Committee of the School of Humanities and Social Sciences at Beijing Institute of Technology with written informed consent from all subjects. All subjects gave written informed consent in accordance with the Declaration of Helsinki. The protocol was approved by the Ethics Committee of the School of Humanities and Social Sciences at Beijing Institute of Technology.

## Author Contributions

XJ designed the study. YZ performed the interviews, analyzed the data, and wrote the manuscript.

## Conflict of Interest Statement

The authors declare that the research was conducted in the absence of any commercial or financial relationships that could be construed as a potential conflict of interest.

## References

[B1] Bonanno G. A. Papa A. Lalande K. Zhang N. Noll J. G. (2005). Grief processing and deliberate grief avoidance: a prospective comparison of bereaved spouses and parents in the United States and the People’s Republic of China. *J. Consult. Clin. Psychol.* 73 86–98. 10.1037/0022-006X.73.1.86 15709835

[B2] Bowen M. (1978). *Family Therapy in Clinical Practice.* Lanham, MD: Rowman & Littlefield Publishers.

[B3] Braun V. Clarke V. (2006). Using thematic analysis in psychology. *Qual. Res. Psychol.* 3 77–101. 10.1191/1478088706qp063oa

[B4] Braun V. Clarke V. (2012). “Thematic analysis,” in *APA Handbook of Research Methods in Psychology: Research Designs* ed. Cooper H. (Washington, DC: American Psychological Association) 57–91.

[B5] Cacciatore J. (2012). “A mindfulness guide through grief,” in *Techniques of Grief Therapy: Creative Practices for Counseling the Bereaved* ed. Neimeyer R. A. (New York, NY: Routledge) 67–75.

[B6] Chen E. (2014). Rebuilding social support network: discussion on the self-organisation and formation of mechanisms for the Shidu group—based on two cases in Shanghai. *Soc. Sci. Beijing* 11 55–60.

[B7] Chinese Academy of Social Sciences (2012). *Contemporary Chinese Dictionary.* Beijing: Commercial Press.

[B8] Detmer C. M. Lamberti J. W. (1991). Family grief. *Death Stud.* 15 363–374. 10.1080/07481189108252441

[B9] Fang S. G. (2015). On the social isolation of those who have lost their only child from the perspective of social exclusion theory. *J. Jiangsu Univ.* 3 73–78.

[B10] Fei X. T. (2012). *From the Soil.* Beijing: Peking University Press.

[B11] Gorer G. (1965). *Death, Grief, and Mourning.* New York, NY: Doubleday.

[B12] Guo N. X. Liu Y. Gai G. Q. Ma L. Ma C. (2017). Relation between psychological demand and social function in corps old people: intermediation of social support availability. *Mod. Prev. Med.* 44 1088–1091.

[B13] He L. Tang X. F. Wang J. P. (2017). Life and death connection: a qualitative study on the continuing bonds for parents who lost their only child. *Chin. J. Clin. Psychol.* 25 697–703.

[B14] He L. Tang X. F. Zhu Z. Y. Wang J. P. (2014). Great pain: qualitative research on grief reactions of the parents who lost their only child. *Chin. J. Clin. Psychol.* 22 792–798.

[B15] Herman J. (1992). *Trauma and Recovery.* New York, NY: Basic Books.

[B16] Hou X. L. (2015). Emotional interpretation of people who lost an only child from the perspective of shame theory. *Seeker* 3 19–23.

[B17] Jia X. M. (2005). Memorial ceremony and psychoanalysis—grief reaction of loss. *Chin. Ment. Health J.* 19 569–571.

[B18] Jia X. M. (2010). The function of mental remedy of funeral rites after disasters. *Neural Inj. Funct. Reconstr.* 5 250–252.

[B19] Kissane D. W. Bloch S. (1994). Family grief. *Br. J. Psychiatry* 164 728–740. 10.1192/bjp.164.6.728 7952979

[B20] Liang S. M. (2011). *The Essentials of Chinese Culture.* Shanghai: Shanghai People’s Publishing House.

[B21] Markus H. R. Kitayama S. (1991). Culture and the self: implications for cognition, emotion, and motivation. *Psychol. Rev.* 98 224–253. 10.1037/0033-295X.98.2.224

[B22] Michon B. Balkou S. Hivon R. Cyr C. (2003). Death of a child: parental perception of grief intensity - end-of-life and bereavement care. *Paediatr. Child Health* 8 363–366. 10.1093/pch/8.6.363 20052330 PMC2795457

[B23] Neimeyer R. A. (2012). The (half) truth about grief. *Illn. Crises Loss* 20 389–395. 10.2190/IL.20.4.g

[B24] Olivas L. (2013). *After-Death Communication: A Parent who Has Lost a Child.* Doctor’s thesis, Walden University Minneapolis, MN.

[B25] Rando T. A. (1983). An investigation of grief and adaptation in parents whose children have died from cancer. *J. Paediatr. Psychol.* 8 3–20. 10.1093/jpepsy/8.1.3 6842349

[B26] Rolls E. M. (2007). *Containing Grief: Ambiguities and Dilemmas in the Emotional Work of UK Childhood Bereavement Services.* Ph.D. thesis, University of Gloucestershire Cheltenham.

[B27] Ronen R. Packman W. Field N. P. Davies B. Kramer R. Long J. K. (2010). The relationship between grief adjustment and continuing bonds for parents who have lost a child. *Omega* 60 1–31. 10.2190/OM.60.1.a 20039529

[B28] Schwartz S. H. (1990). Individualism-collectivism: critique and proposed refinements. *J. Cross Cult. Psychol.* 21 139–157. 10.1177/0022022190212001

[B29] Shang Z. L. (2016). *Study on PTSD Caused by Major Acute Stress (Losing an only Child) and its Influencing Factors.* Master’s thesis, Second Military Medical University Shanghai.

[B30] Steinke I. (2004). “Quality criteria in qualitative research,” in *A Companion to Qualitative Research* eds Flick U. Kardorff E. V. Steinke I. (London: Sage) 184–190.

[B31] Stroebe M. S. Hansson R. O. Stroebe W. Schut H. (2001). “Introduction: concepts and issues in contemporary research on bereavement,” in *Handbook of Bereavement Research: Consequences, Coping, and Care* eds Stroebe M. S. Hansson R. O. Stroebe W. Schut H. (Washington, DC: American Psychological Association) 3–22.

[B32] Symington J. Symington N. (1996). *The Clinical Thinking of Wilfred Bion.* London: Routledge.

[B33] Tedeschi R. G. Calhoun L. G. (2004). Posttraumatic growth: conceptual foundations and empirical evidence. *Psychol. Inq.* 15 1–18. 10.1207/s15327965pli1501_01

[B34] Wang D. Cao X. Yang P. Liu R. (2017). Mental health and the relating factors among Chinese older adults who lost their only children. *Innov. Ageing* 1:339 10.1093/geroni/igx004.1245

[B35] Wang G. Z. (2013). Study on the total number of deceased only children and changing trends. *Chin. J. Popul. Sci.* 1 57–65.

[B36] Wang G. Z. (2016). Research using computer simulation for the population size, age structure and developing trend of women who lost an only child. *Popul. Econ.* 5 1–11.

[B37] World Health Organisation (2017). *Prolonged Grief Disorder [Online]*. Available at: https://icd.who.int/dev11/l-m/en#/http://id.who.int/icd/entity/1183832314 [accessed October 31, 2017].

[B38] Wu W. L. (2011). Understanding and handling loss in Chinese culture. *J. Xihua Univ.* 30 5–7.

[B39] Xiu D. Maercker A. Woynar S. Geirhofer B. Yang Y. Jia X. (2016). Features of prolonged grief symptoms in chinese and Swiss bereaved parents. *J. Nerv. Ment. Dis.* 204 693–701. 10.1097/NMD.0000000000000539 27253073

[B40] Xu X. J. (2014). The path, types and social risks of the marginalisation of parents who lost an only child—from the perspective of the relationship between individual and group. *J. Huazhong Norm. Univ.* 53 22–30.

[B41] Xu Y. Herrman H. Bentley R. Tsutsumi A. Fisher J. (2014). Effect of having a subsequent child on the mental health of women who lost a child in the 2008 Sichuan earthquake: a cross-sectional study. *Bull. World Health Organ.* 92 348–355. 10.2471/BLT.13.124677 24839324 PMC4007123

[B42] Yang G. S. (2004). *The Psychology and Behaviour of Chinese People: An Indigenous Study.* Beijing: China Renmin University Press.

[B43] Yang Q. K. (2007). *Religion in Chinese Society.* Shanghai: Shanghai People’s Publishing House.

[B44] Yang Z. Y. (1992). *People’s Outlook on Life and Death in Southwest China.* Kunming: Yunnan Education Publishing House.

[B45] Yang Z. Y. (2000). *Familism Culture and Chinese Culture.* Kunming: Yunnan University Press.

[B46] Yao S. Y. Wang A. N. Zhang W. Luo Y. H. Zhang J. P. (2016). Social support and subject well-being of people who have lost as only child: the mediating role of self-efficacy. *Chin. J. Behav. Med. Sci.* 25 1114–1117.

[B47] Zheng Y. Lawson T. R. (2014). Identity reconstruction asshiduers: narratives from Chinese older adults who lost their only child. *Int. J. Soc. Welf.* 24 399–406. 10.1111/ijsw.12139

